# Trends in the Incidence and Survival Rates of Colorectal Signet-Ring Cell Carcinoma in the South Korean Population: Analysis of the Korea Central Cancer Registry Database

**DOI:** 10.3390/jcm10184258

**Published:** 2021-09-20

**Authors:** Ji-Hoon Kim, Hyunil Kim, Jin Woo Kim, Hee Man Kim

**Affiliations:** 1 Department of Medicine, Yonsei University Wonju College of Medicine, Wonju 26426, Korea; jh12111000@hanmail.net; 2 Department of Internal Medicine, Yonsei University Wonju College of Medicine, Wonju 26426, Korea; kimhyunil@empas.com (H.K.); bluebloood@naver.com (J.W.K.)

**Keywords:** colon, rectum, signet-ring cell carcinoma, incidence, survival

## Abstract

Objective: Signet-ring cell carcinoma (SRCC) is a rare histopathological subtype of colorectal cancer (CRC) constituting approximately 1% of CRC cases. This study analyzed the incidence and survival rates of colorectal SRCC. Methods: We analyzed the incidence and survival rates of colorectal SRCCs based on patients’ data of the Korea Central Cancer Registry. Results: The age-standardized incidence rates of colon and rectum SRCC in 2017 were 0.17 and 0.07 individuals per 100,000, respectively. Between 1993 and 2017, the 1-, 2-, 3-, 4-, and 5-year relative survival rates of patients with colon SRCC were 65.6%, 49.0%, 38.9%, 34.9%, and 33.0%, respectively, while those of patients with rectum SRCC were 69.6%, 47.8%, 38.5%, 32.8%, and 29.4%, respectively. According to the Surveillance, Epidemiology, and End Results summary stages, the 5-year relative survival rates of colon SRCC between 1993 and 2017 were 70.4% for the localized stage, 41.0% for the regional stage, and 7.0% for the distant stage, while those for rectum SRCC were 60.7%, 34.4, and 3.3%, respectively. Conclusions: Although the incidence of colorectal SRCC is extremely low in South Korea, it has been increasing in recent decades. As the prognosis of colorectal SRCC is extremely poor; clinicians should be aware of the differential diagnosis of SRCC in colorectal cancer cases.

## 1. Introduction

Colorectal cancer (CRC) is a significant global health burden, ranking third in cancer-associated mortality among all types of malignancies [1,2,3]. The clinical features and prognosis of CRC are highly heterogeneous in histopathological subtypes [4,5,6,7]. Signet-ring cell carcinoma (SRCC) is a very rare histopathological subtype of CRC that constitutes approximately 1% of CRC cases [8,9]. SRCC is defined as carcinoma with >50% of signet-ring cells, characterized by intracytoplasmic mucin that displaces the nucleus to the periphery [10,11,12]. SRCCs mostly originate from undifferentiated stem cells of the colorectal mucosa and often show poor differentiation, diffuse infiltration, rapid growth, and high metastatic frequency [13,14]. SRCCs have an extremely poor prognosis as they are generally detected at an advanced stage and are in an unresectable state [12,15,16,17,18]. Despite its rarity, colorectal SRCC is clinically crucial because it is associated with a poor survival outcome.

To date, limited population-based studies on the incidence and survival rates of colorectal SRCCs have been reported, possibly because of its low incidence. All previous studies used Western population databases, generally the Surveillance, Epidemiology, and End Results (SEER) database, which is the largest cancer dataset in the United States [8,19,20,21]. However, there is a survival disparity between Western and Asian CRC owing to differences in biology and socioeconomic status; hence, investigation of the incidence and survival rates of colorectal SRCC in Asians is necessary [22]. The present nationwide population-based study analyzed Korea Central Cancer Registry (KCCR) data between 1999 and 2017.

## 2. Materials and Methods

The Korean National Cancer Incidence Database (KNCIDB), a nationwide population-based database of cancer occurrence, was constructed by the KCCR of the National Cancer Center (Goyang, South Korea) [23]. We obtained the necessary approval from the KCCR. The KNCIDB classifies cancer according to the International Classification of Diseases for Oncology, third edition (ICD-O-3) [11]. We used ICD code 8490/3 to retrieve data on SRCC from the KNCIDB. ICD codes C18 and C19–20 were used to define colon cancer and rectal cancer, respectively. These methods are used widely in other studies [21,24,25]. That is, the diagnosis in the Korean National Cancer Registry is based on pathology.

A total of 10,780 patients who were diagnosed with colorectal SRCC between 1999 and 2017 were identified in the KNCIDB, which included data on patient age and sex and the SEER summary stages of the tumor. The SEER summary stages, which are used to categorize tumor stage in the KNCIDB, include localized, regional, distant, and unknown stages.

The crude incidence rate was defined as the total number of newly diagnosed cases in a year divided by the mid-year population in South Korea [26]. The age-standardized incidence rate, the weighted average of the age-specific rates, was calculated using Segi’s world standard population [26,27]. The relative survival rate (RSR) was defined as the ratio of the observed survival rate to the expected survival rate. The expected survival rate, defined as the expected mortality of the general population of the same age and sex, was calculated using the Ederer II method [26,28]. The 5-year RSR was calculated for the following periods: 1993–1995, 1996–2000, 2001–2005, 2006–2010, 2011–2015, and 2013–2017. The 10-year RSR was calculated for the following periods: 1993–1995, 1996–2000, 2001–2005, 2006–2010, and 2008–2012. SAS ver. 9.4 (SAS Institute Inc., Cary, NC, USA) was used for statistical analysis.

### Ethics Statement

This study was approved by the Institutional Review Board of Wonju Severance Christian Hospital (CR319145), which waived the requirement for informed consent from the patients.

## 3. Results

### 3.1. Incidence of Colon SRCC

**Figure 1:**
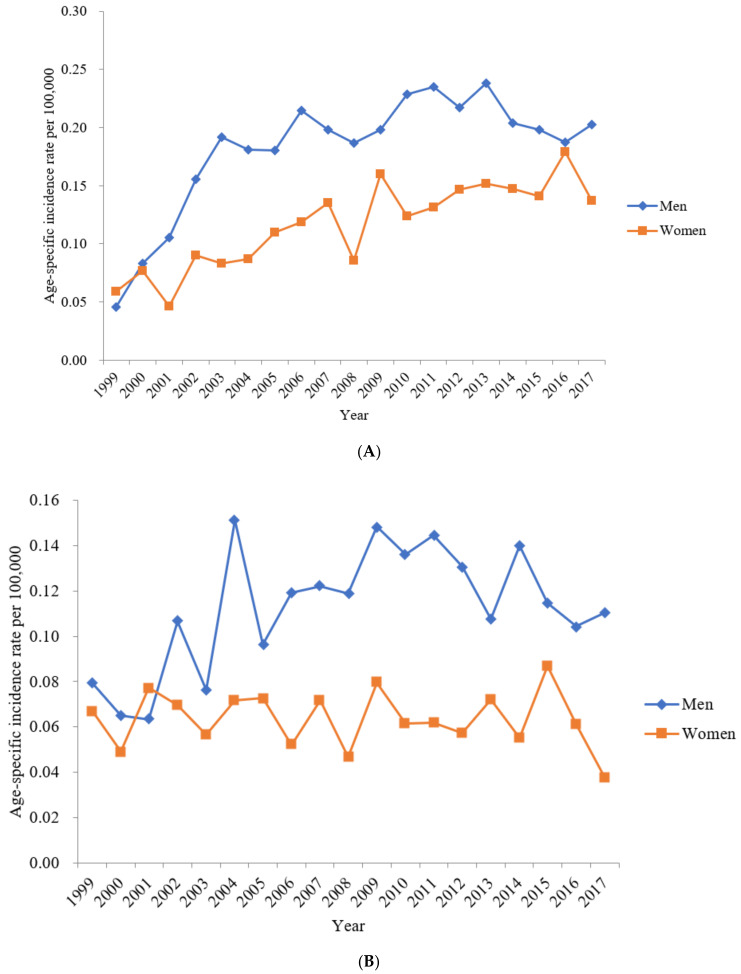
The age-standardized incidence rate of colon and rectum signet-ring cell carcinoma. (**A**) The age-standardized incidence rate of colon signet-ring cell carcinoma per 100,000 individuals between 1999 and 2017. (**B**) The age-standardized incidence rate of rectum signet-ring cell carcinoma per 100,000 individuals between 1999 and 2017.

The number of patients diagnosed with colon SRCC in South Korea gradually increased from 1999 (24 cases among 46.4 million individuals) to 2017 (116 cases among 51.4 million individuals) (Table 1). The crude and age-standardized incidence rates increased from 1999 to 2017. In 2017, the age-standardized incidence rates of colon SRCC in men and women were 0.20 and 0.14 per 100,000 individuals, respectively. Men had a slightly higher age-standardized incidence rate of colon SRCC than women (Figure 1A).

### 3.2. Incidence of Rectum SRCC

The numbers of patients diagnosed with rectum SRCC in South Korea were similar from 1999 (34 cases among 46.4 million individuals) to 2017 (52 cases among 51.4 million individuals) (Table 2). The crude and age-standardized incidence rates were similar from 1999 to 2017. In 2017, the age-standardized incidence rates of rectum SRCC in men and women were 0.11 and 0.04 per 100,000 individuals, respectively. Men had a slightly higher age-standardized incidence rate of rectum SRCC than women (Figure 1B).

### 3.3. SEER Summary Stages of Colon SRCC between 2005 and 2017

**Figure 2:**
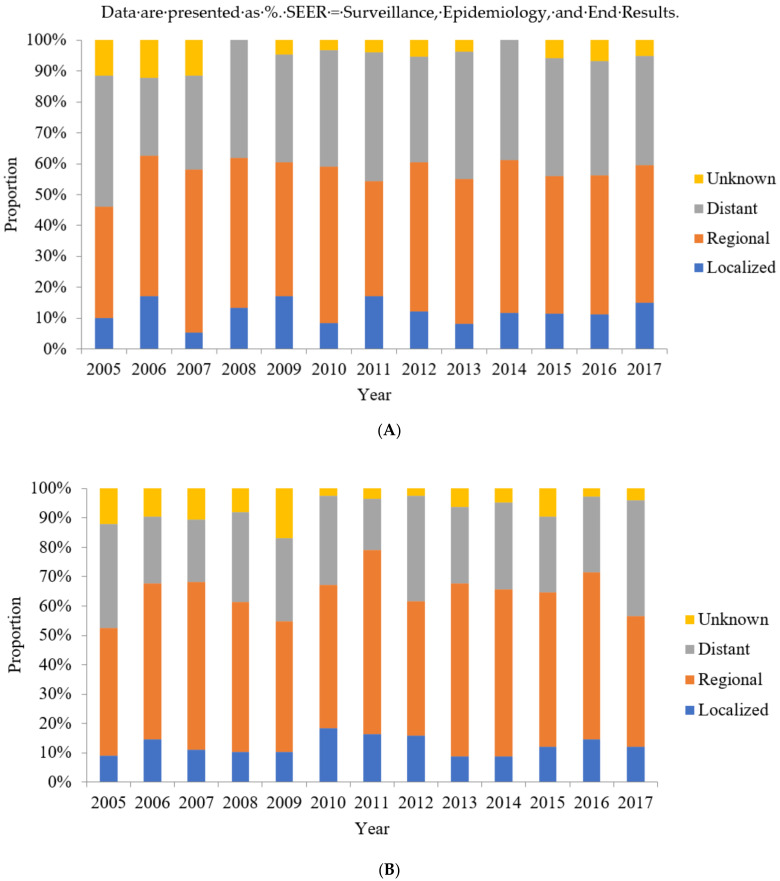
The proportion of Surveillance, Epidemiology, and End Results summary stage of patients with colon and rectum signet-ring cell carcinoma between 2005 and 2017. (**A**) The proportion of Surveillance, Epidemiology, and End Results summary stage of patients with colon signet-ring cell carcinoma between 2005 and 2017. (**B**) The proportion of Surveillance, Epidemiology, and End Results summary stage of patients with rectum signet-ring cell carcinoma between 2005 and 2017.

Based on the SEER summary staging, regional-stage cases accounted for the largest proportion of cases diagnosed with colon SRCC between 2005 and 2017 (Table 3). Among all colon SRCC cases, the proportions of cases with regional- and distant-stage disease were relatively constant (Figure 2A). Moreover, the proportions of cases with regional plus distant stage were constant, accounting for almost all colon SRCC cases (range, 70.6–88.4%).

### 3.4. SEER Summary Stages of Rectum SRCC between 2005 and 2017

Regional-stage disease accounted for the largest proportion of cases diagnosed with rectum SRCC between 2005 and 2017 (Table 4). Among all rectal SRCC cases, the proportions of regional- and distant-stage cases were relatively constant (Figure 2B). Moreover, the proportion of regional plus distant SEER summary stage cases were constant, accounting for almost all rectum SRCC cases (range, 73.0–86.4%).

### 3.5. RSRs of Colon SRCC

**Figure 3:**
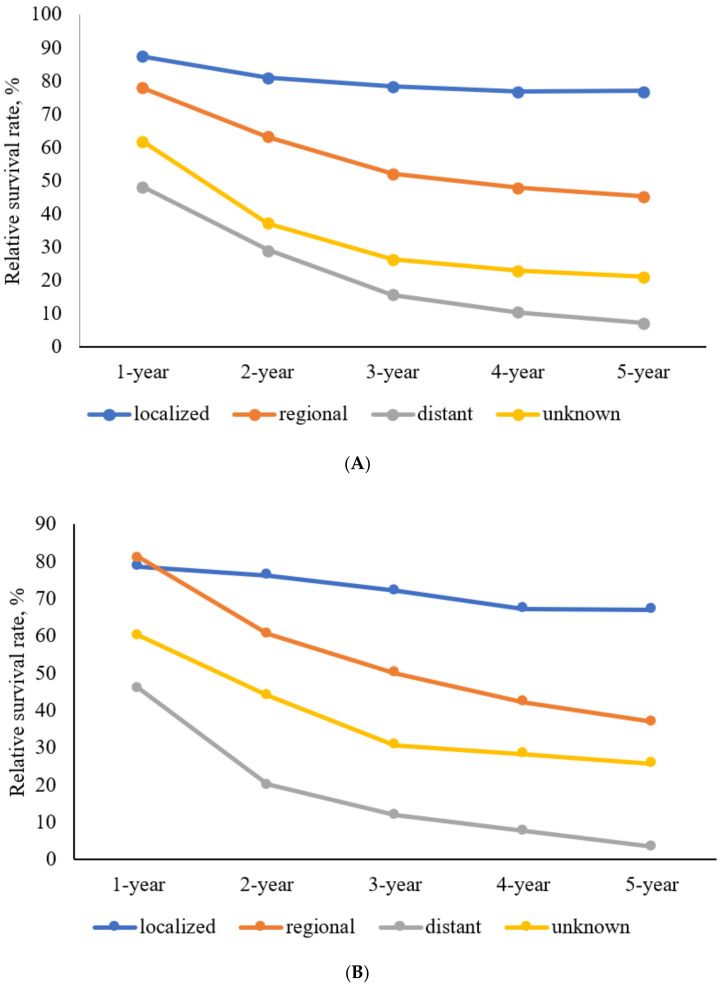
Relative survival rate of patients with colorectum signet-ring cell carcinoma between 1993 and 2017. (**A**) Relative survival rate of patients with colon signet-ring cell carcinoma between 1993 and 2017. (**B**) Relative survival rate of patients with rectum signet-ring cell carcinoma between 1993 and 2017.

Between 1993 and 2017, the 1-, 2-, 3-, 4-, and 5-year RSRs of patients with colon SRCC were 65.6%, 49.0%, 38.9%, 34.9%, and 33.0%, respectively, and 48.4%, 29.2%, 15.7%, 10.4%, and 7.4%, respectively, among those with distant SEER summary stage (Figure 3A). Among patients with various SEER summary stages of colon SRCC, men showed a slightly better 5-year RSR than women (Table 5).

**Figure 4:**
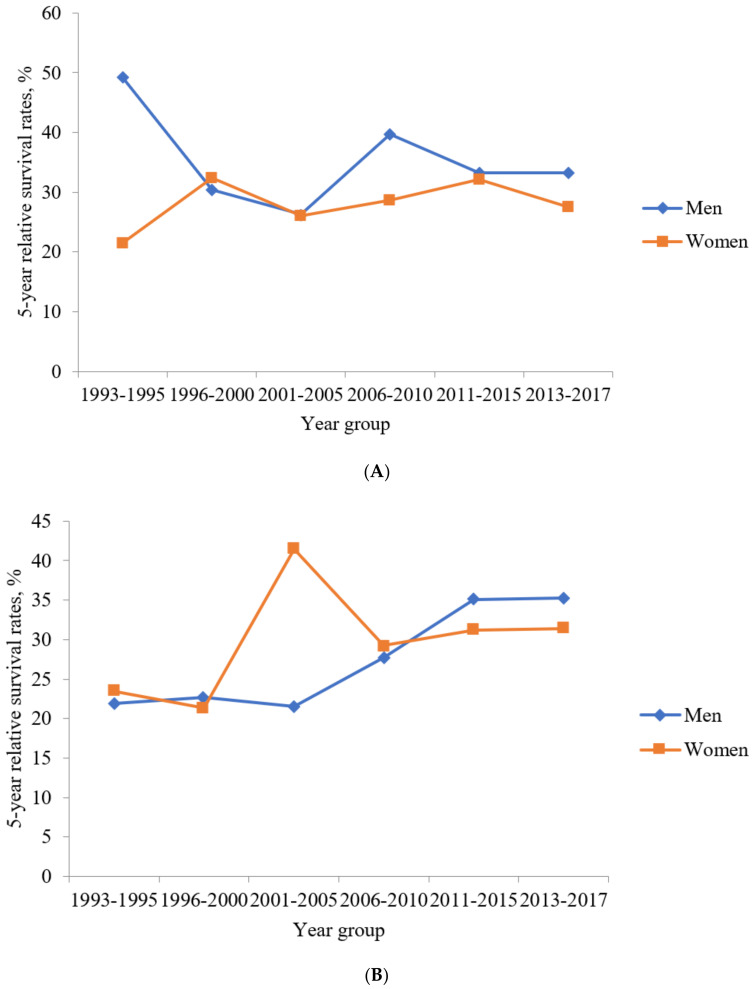
The five-year relative survival rates of patients with colorectal signet-ring cell carcinoma in different time periods (1996–2000, 2001–2005, 2006–2010, 2011–2015, 2013–2017). (**A**) The five–year relative survival rates of patients with colon signet-ring cell carcinoma in different time periods (1996–2000, 2001–2005, 2006–2010, 2011–2015, 2013–2017). (**B**) The five–year relative survival rates of patients with rectum signet-ring cell carcinoma in different time periods (1996–2000, 2001–2005, 2006–2010, 2011–2015, 2013–2017).

**Figure 5:**
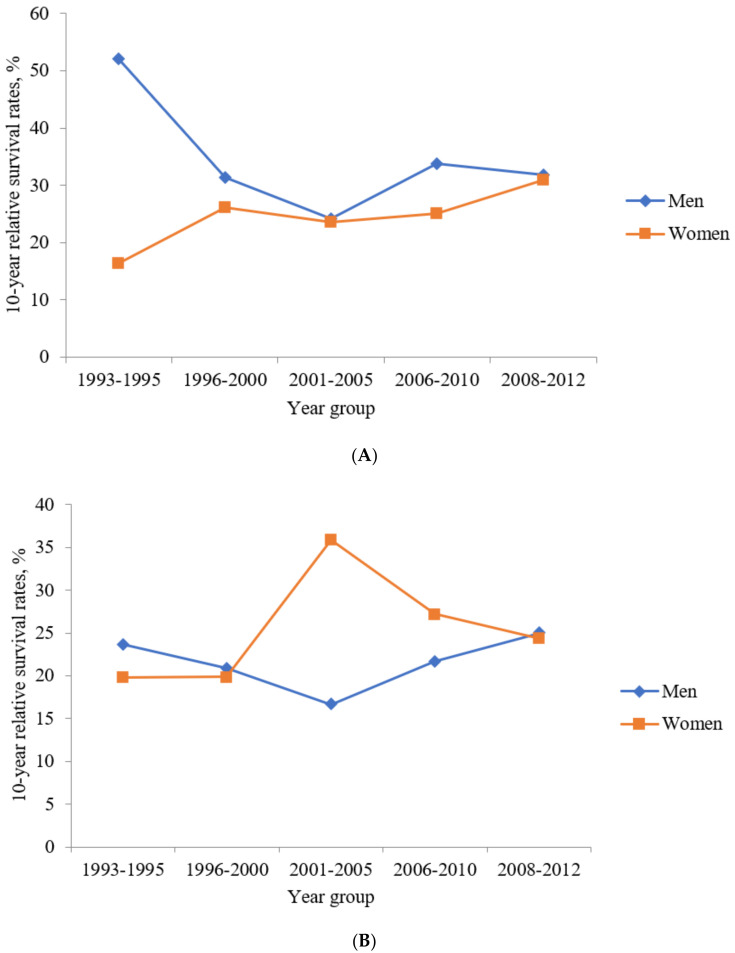
The ten-year relative survival rates of patients with colorectal signet-ring cell carcinoma in different time periods (1996–2000, 2001–2005, 2006–2010, 2008–2012). (**A**) The ten–year relative survival rates of patients with colon signet-ring cell carcinoma in different time periods (1996–2000, 2001–2005, 2006–2010, 2008–2012). (**B**) The ten–year relative survival rates of patients with rectum signet-ring cell carcinoma in different time periods (1996–2000, 2001–2005, 2006–2010, 2008–2012).

The 5-year RSRs were 38.6%, 31.3%, 26.2%, 35.4%, 32.8%, and 30.8% during 1993–1995, 1996–2000, 2001–2005, 2006–2010, 2011–2015, and 2013–2017, respectively. The 10-year RSRs were 38.2%, 29.1%, 24.0%, 30.4%, and 31.3% during 1993–1995, 1996–2000, 2001–2005, 2006–2010, and 2008–2012, respectively. The 5-year RSRs gradually decreased in men but gradually increased in women during 1993–1995 and 2013–2017 (Figure 4A). Similarly, while the 10-year RSRs gradually decreased in men, it gradually increased in women during 1993–1995 and 2008–2012 (Figure 5A). The 5-year RSRs were higher in men than in women in every year group except for 1996–2000.

### 3.6. RSRs of Rectum SRCC

Between 1993 and 2017, the 1-, 2-, 3-, 4-, and 5-year RSRs of patients with rectum SRCC were 69.6%, 47.8%, 38.5%, 32.8%, and 29.4%, respectively, while those for patients with distant SEER summary stage rectum SRCC were 46.1%, 20.2%, 12.0%, 7.7%, and 3.4%, respectively (Figure 3B). Among patients with rectum SRCC, men exhibited a better 5-year RSR than women in the localized and distant but not the regional stage (Table 6).

The 5-year RSRs were 22.6%, 22.1%, 30.1%, 28.2%, 33.8%, and 34.0% during 1993–1995, 1996–2000, 2001–2005, 2006–2010, 2011–2015, and 2013–2017, respectively. The 10-year RSRs were 21.7%, 20.4%, 25.0%, 23.6%, and 24.4% during 1993–1995, 1996–2000, 2001–2005, 2006–2010, and 2008–2012, respectively. The 5-year RSRs were the lowest in men but the highest in women during 2001–2005 (Figure 4B). In addition, the 10-year RSRs were the lowest in men but the highest in women during 2001–2005 (Figure 5B). The 5-year RSRs were higher in men than in women during 1996–2000, 2011–2015, and 2013–2017. However, the 5-year RSRs were lower in men than those in women during 1993–1995, 2001–2005, and 2006–2010.

## 4. Discussion

Our study evaluated the nationwide incidence and survival rates of colorectal SRCCs in South Korea, the incidence of which was extremely low. In 2017, only 168 individuals of the 51.4 million population in South Korea were diagnosed with colorectal SRCC. In addition, the results of our study revealed that the age-standardized incidence rate of colon SRCC has gradually increased, while that of rectum SRCC has remained stable, from 1999 to 2017.

A population-based study conducted in the United States reported that 4740 and 936 patients were diagnosed with colon SRCC and rectum SRCC between 2000 and 2014, respectively, with the highest age-adjusted incidence rates of 0.41 and 0.075 per 100,000 individuals, respectively, for colon SRCC and rectum SRCC [19]. In our study, the highest age-standardized incidence rates during 2000–2014 were 0.19 per 100,000 individuals for colon SRCC and 0.10 per 100,000 individuals for rectum SRCC. The age-adjusted incidence rates for colon SRCC in the United States gradually decreased from 2000 to 2014, while those in South Korea have gradually increased [19]. We must be careful when comparing and interpreting these incidences. This is because the definitions of terms used in the studies are different. In addition, it should be kept in mind that Korea cannot be directly compared to the multiethnic United States because it is relatively monoethnic.

The National Cancer Institute’s SEER database from 1975–2016 showed that SRCC comprised 1.0% of all cases of colorectal cancer [24]. Other studies reported that SRCC consist of 0.1–2.6% of CRC [18,29]. In our study, SRCC comprised 0.59% (168 patients) of all colorectal cancer (28,111 patients) in 2017, and our results fall within this range. 

**Figure 6:**
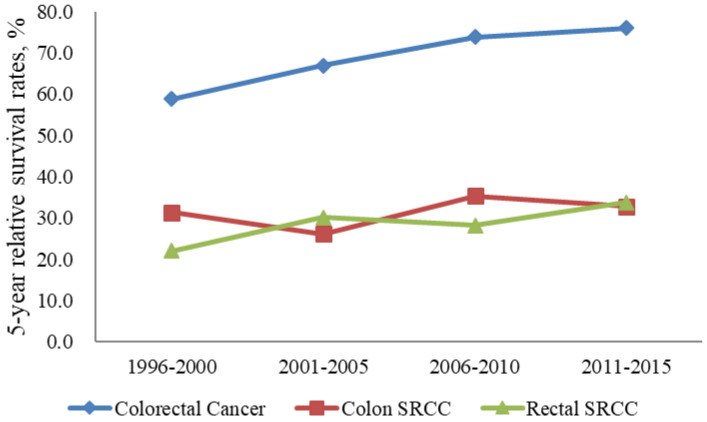
Five-year relative survival rate of all colorectal cancer and SRCC from 1996 to 2015.

Colorectal SRCC is associated with a poor prognosis as it is generally diagnosed at an advanced stage [12,15,16,17,18]. In our study, the proportions of cases with distant metastasis were the second largest among colon SRCC and rectum SRCC cases. The 5-year RSRs of patients in South Korea with colon SRCC and rectum SRCC were 33.0% and 29.4%, respectively, between 1993 and 2017. In the United States, the 5-year RSRs of patients with colon SRCC and rectum SRCC were 28.6% and 21.1%, respectively, between 1992 and 2000 [8]. In our study, the 5-year RSR of colon SRCC was higher than that of rectum SRCC, consistent with the findings of other studies [8,20]. However, although the survival rate is poor, the survival rate is increasing compared to the past (Figure 6). This is the same phenomenon as the increase in colorectal cancer survival rate in South Korea.

Because SRCC of colorectal cancer is so rare, therapeutic guidelines remain undetermined due to a lack of large randomized controlled trials. To date, surgery is considered as the most important option of treatment [21]. One population-based study showed that adjuvant chemotherapy was beneficial in stage III SRCC patients [4]. In metastatic colorectal SRCC, one study reported a better survival of chemotherapy in patients [30], but other studies reported poor response of chemotherapy in colorectal SRCC [31,32]. In SRCC of rectal cancer, neoadjuvant chemoradiotherapy could give rise to good therapeutic response [33]. Further prospective randomized controlled trials are necessary to make the optimal therapeutic strategies.

There is a disparity in the incidence and survival rates of colorectal SRCCs between men and women. In our study, men had higher age-standardized incidence rates of both colon and rectal SRCCs compared to those in women. In addition, colon SRCC showed a trend of higher 5- and 10-year RSRs in men than in women, contrary to the findings of other studies reporting a lower hazard ratio of death in women than in men [8,19]. Further, some studies showed no significant difference in the hazard ratios of death between women and men [20,21]. There are research papers that report that hormone or genetic differences cause these results [24]. However, different countries report different results. Although several studies revealed gender differences, the results did not show a common trend. One study of Surveillance, Epidemiology, and End Results (SEER) database from 2004 until 2015 showed that among qualified postoperative patients, there were more men than women [34]. One study showed that, in SEER database between 2004 and 2015, numbers of male were 1677 (51.16%) and those of female was 1601 (48.84%) [21]. In SEER 1975–2016, numbers of male were 2276 (49.6%), and those of female were 2310 (50.4%) [35].

Our nationwide study included a large number of patients with colorectal SRCC (*n* = 10,780). This made it possible to comprehensively investigate the trends in the incidence and long-term survival outcomes of patients with colorectal SRCCs in South Korea. In addition, we analyzed all C18 (colon) and C19-20 (rectum) cases. Despite these strengths, our study has some limitations. First, data on clinical information, comorbidities, family history, and molecular genetic profiles were not available. Second, data on treatment modalities, including chemotherapy or radiotherapy, were not available. Lastly, data on several factors affecting survival that may have provided further insights, such as the distant metastasis site, progression-free survival, and recurrence-free survival, were not available.

In conclusion, although the incidence of colorectal SRCC is extremely low in South Korea, it has been increasing in recent decades. The prognosis is extremely poor overall, and especially so in advanced stages of colorectal SRCC. Hence, clinicians should be aware of the differential diagnosis of SRCCs in colorectal cancer cases. As the KNCIDB does not include clinical information, further population-based studies are needed to thoroughly investigate the survival rates of colorectal SRCCs in Asian populations.

## Figures and Tables

**Table 1:** Incidence of colon signet-ring cell carcinoma in South Korea between 1999 and 2017.

Year	All (*n*)	Colon (C18) Signet-Ring Cell Carcinoma					
		Crude Incidence Rate ^a^			Age-Standardized Incidence Rate ^a^		
		All	Men	Women	All	Men	Women
1999	24	0.05	0.04	0.06	0.05	0.05	0.06
2000	38	0.08	0.08	0.08	0.08	0.08	0.08
2001	37	0.08	0.10	0.05	0.08	0.11	0.05
2002	62	0.13	0.16	0.10	0.12	0.16	0.09
2003	68	0.14	0.20	0.08	0.13	0.19	0.08
2004	71	0.15	0.19	0.10	0.13	0.18	0.09
2005	79	0.16	0.20	0.13	0.14	0.18	0.11
2006	92	0.19	0.24	0.14	0.17	0.21	0.12
2007	95	0.19	0.22	0.16	0.17	0.20	0.14
2008	79	0.16	0.21	0.11	0.13	0.19	0.09
2009	109	0.22	0.24	0.20	0.18	0.20	0.16
2010	108	0.22	0.28	0.16	0.17	0.23	0.12
2011	116	0.23	0.28	0.18	0.18	0.23	0.13
2012	118	0.23	0.27	0.19	0.18	0.22	0.15
2013	127	0.25	0.30	0.21	0.19	0.24	0.15
2014	117	0.23	0.27	0.19	0.17	0.20	0.15
2015	122	0.24	0.27	0.21	0.17	0.20	0.14
2016	135	0.26	0.26	0.27	0.18	0.19	0.18
2017	116	0.23	0.27	0.19	0.17	0.20	0.14

^a^ Per 100,000 individuals.

**Table 2:** Incidence of rectal signet-ring cell carcinoma in South Korea between 1999 and 2017.

Year	All (*n*)	Rectum (C19–20) Signet-Ring Cell Carcinoma					
		Crude Incidence Rate ^a^			Age-Standardized Incidence Rate ^a^		
		All	Men	Women	All	Men	Women
1999	34	0.07	0.08	0.07	0.07	0.08	0.07
2000	27	0.06	0.06	0.05	0.06	0.07	0.05
2001	35	0.07	0.06	0.08	0.07	0.06	0.08
2002	44	0.09	0.11	0.08	0.09	0.11	0.07
2003	34	0.07	0.08	0.06	0.07	0.08	0.06
2004	59	0.12	0.16	0.08	0.11	0.15	0.07
2005	45	0.09	0.10	0.08	0.08	0.10	0.07
2006	47	0.10	0.13	0.06	0.08	0.12	0.05
2007	56	0.11	0.14	0.09	0.10	0.12	0.07
2008	47	0.10	0.13	0.06	0.08	0.12	0.05
2009	71	0.14	0.18	0.10	0.11	0.15	0.08
2010	62	0.12	0.17	0.08	0.10	0.14	0.06
2011	69	0.14	0.19	0.09	0.10	0.14	0.06
2012	63	0.13	0.17	0.08	0.09	0.13	0.06
2013	61	0.12	0.14	0.10	0.09	0.11	0.07
2014	68	0.13	0.20	0.07	0.10	0.14	0.06
2015	67	0.13	0.16	0.10	0.10	0.11	0.09
2016	59	0.12	0.14	0.09	0.08	0.10	0.06
2017	52	0.10	0.15	0.05	0.07	0.11	0.04

^a^ Per 100,000 individuals.

**Table 3:** SEER summary stages of colon signet-ring cell carcinoma between 2005 and 2017.

Year	Men				Women			
	Localized	Regional	Distant	Unknown	Localized	Regional	Distant	Unknown
2005	10.4	33.3	39.6	16.7	9.7	38.7	45.2	6.5
2006	13.8	46.6	24.1	15.5	20.6	44.1	26.5	8.8
2007	5.5	58.2	23.6	12.7	5.0	47.5	37.5	10.0
2008	18.9	47.2	34.0	0.0	7.7	50.0	42.3	0.0
2009	20.0	41.7	35.0	3.3	14.3	44.9	34.7	6.1
2010	11.6	58.0	29.0	1.4	5.1	43.6	46.2	5.1
2011	21.1	36.6	36.6	5.6	13.3	37.8	46.7	2.2
2012	10.1	43.5	37.7	8.7	14.3	53.1	30.6	2.0
2013	6.7	44.0	44.0	5.3	9.6	50.0	38.5	1.9
2014	13.2	60.3	26.5	0.0	10.2	38.8	51.0	0.0
2015	11.8	48.5	35.3	4.4	11.1	40.7	40.7	7.4
2016	13.6	37.9	42.4	6.1	8.7	52.2	31.9	7.2
2017	13.2	45.6	35.3	5.9	16.7	43.8	35.4	4.2

**Table 4:** SEER summary stages of rectum signet-ring cell carcinoma between 2005 and 2017.

Year	Men				Women			
	Localized	Regional	Distant	Unknown	Localized	Regional	Distant	Unknown
2005	8.0	52.0	36.0	4.0	10.0	35.0	35.0	20.0
2006	9.4	59.4	18.8	12.5	20.0	46.7	26.7	6.7
2007	17.1	57.1	14.3	11.4	4.8	57.1	28.6	9.5
2008	6.1	45.5	39.4	9.1	14.3	57.1	21.4	7.1
2009	8.9	46.7	37.8	6.7	11.5	42.3	19.2	26.9
2010	20.9	55.8	18.6	4.7	15.8	42.1	42.1	0.0
2011	19.1	61.7	17.0	2.1	13.6	63.6	18.2	4.5
2012	11.6	46.5	37.2	4.7	20.0	45.0	35.0	0.0
2013	5.6	50.0	36.1	8.3	12.0	68.0	16.0	4.0
2014	12.0	64.0	20.0	4.0	5.6	50.0	38.9	5.6
2015	4.9	51.2	36.6	7.3	19.2	53.8	15.4	11.5
2016	10.8	59.5	24.3	5.4	18.2	54.5	27.3	0.0
2017	2.6	60.5	28.9	7.9	21.4	28.6	50.0	0.0

Data are presented as %. SEER = Surveillance, Epidemiology, and End Results.

**Table 5:** Relative survival rates of patients with colon signet-ring cell carcinoma between 1993 and 2017.

	SEER Stage	Relative Survival Rates (%)				
		1-Year	2-Year	3-Year	4-Year	5-Year
Men	Localized	88.5	81.8	79.5	78.1	79.3
	Regional	78.2	65.4	54.0	49.3	46.5
	Distant	48.2	30.3	17.1	10.6	8.5
	Unknown	62.1	37.8	31.0	25.9	23.3
Women	Localized	86.0	79.8	76.5	75.1	73.4
	Regional	77.7	60.2	49.7	46.2	44.0
	Distant	48.6	27.9	14.1	10.3	5.9
	Unknown	61.4	36.3	17.1	17.3	17.4

SEER = Surveillance, Epidemiology, and End Results.

**Table 6:** Relative survival rates of patients with rectum signet-ring cell carcinoma between 1993 and 2017.

	SEER Stage	Relative Survival Rates (%)				
		1-Year	2-Year	3-Year	4-Year	5-Year
Men	Localized	83.4	78.5	73.3	67.1	68.3
	Regional	80.5	59.3	48.8	41.6	36.0
	Distant	46.7	21.0	11.3	8.0	4.0
	Unknown	48.0	40.5	30.7	25.4	25.7
Women	Localized	72.0	73.2	71.0	68.2	65.2
	Regional	82.8	63.6	52.6	44.0	39.2
	Distant	44.7	18.6	13.0	7.1	2.4
	Unknown	78.8	50.0	32.0	32.9	27.2

SEER = Surveillance, Epidemiology, and End Results.

## Data Availability

The Korean National Cancer Incidence Database (https://ncc.re.kr, accessed on 11 September 2021).

## References

[1] Byeon J.S., Yang S.K., Kim T.I., Kim W.H., Lau J.Y., Leung W.K., Fujita R., Makharia G.K., Abdullah M., Hilmi I. (2007). Colorectal neoplasm in asymptomatic Asians: A prospective multinational multicenter colonoscopy survey. Gastrointest. Endosc..

[2] Sung J.J., Lau J.Y., Goh K.L., Leung W.K., Asia Pacific Working Group on Colorectal Cancer (2005). Increasing incidence of colorectal cancer in Asia: Implications for screening. Lancet Oncol..

[3] Siegel R.L., Miller K.D., Goding Sauer A., Fedewa S.A., Butterly L.F., Anderson J.C., Cercek A., Smith R.A., Jemal A. (2020). Colorectal cancer statistics, 2020. CA Cancer J. Clin..

[4] Hugen N., Verhoeven R.H., Lemmens V.E., van Aart C.J., Elferink M.A., Radema S.A., Nagtegaal I.D., de Wilt J.H. (2015). Colorectal signet-ring cell carcinoma: Benefit from adjuvant chemotherapy but a poor prognostic factor. Int. J. Cancer.

[5] Mizushima T., Nomura M., Fujii M., Akamatsu H., Mizuno H., Tominaga H., Hasegawa J., Nakajima K., Yasumasa K., Yoshikawa M. (2010). Primary colorectal signet-ring cell carcinoma: Clinicopathological features and postoperative survival. Surg. Today.

[6] Nitsche U., Zimmermann A., Spath C., Muller T., Maak M., Schuster T., Slotta-Huspenina J., Kaser S.A., Michalski C.W., Janssen K.P. (2013). Mucinous and signet-ring cell colorectal cancers differ from classical adenocarcinomas in tumor biology and prognosis. Ann. Surg..

[7] Thota R., Fang X., Subbiah S. (2014). Clinicopathological features and survival outcomes of primary signet ring cell and mucinous adenocarcinoma of colon: Retrospective analysis of VACCR database. J. Gastrointest. Oncol..

[8] Kang H., O’Connell J.B., Maggard M.A., Sack J., Ko C.Y. (2005). A 10-year outcomes evaluation of mucinous and signet-ring cell carcinoma of the colon and rectum. Dis. Colon Rectum.

[9] Hyngstrom J.R., Hu C.Y., Xing Y., You Y.N., Feig B.W., Skibber J.M., Rodriguez-Bigas M.A., Cormier J.N., Chang G.J. (2012). Clinicopathology and outcomes for mucinous and signet ring colorectal adenocarcinoma: Analysis from the National Cancer Data Base. Ann. Surg. Oncol..

[10] Borger M.E., Gosens M.J., Jeuken J.W., van Kempen L.C., van de Velde C.J., van Krieken J.H., Nagtegaal I.D. (2007). Signet ring cell differentiation in mucinous colorectal carcinoma. J. Pathol..

[11] Bosman F.T., Carneiro F., Hruban R.H., Theise N.D. (2010). WHO Classification of Tumours of the Digestive System.

[12] Messerini L., Palomba A., Zampi G. (1995). Primary signet-ring cell carcinoma of the colon and rectum. Dis. Colon Rectum.

[13] Gopalan V., Smith R.A., Ho Y.H., Lam A.K. (2011). Signet-ring cell carcinoma of colorectum--current perspectives and molecular biology. Int. J. Colorectal. Dis..

[14] Hugen N., van de Velde C.J.H., de Wilt J.H.W., Nagtegaal I.D. (2014). Metastatic pattern in colorectal cancer is strongly influenced by histological subtype. Ann. Oncol..

[15] Chen J.S., Hsieh P.S., Hung S.Y., Tang R., Tsai W.S., Changchien C.R., Lin P.Y., Wang J.Y., Yeh C.Y. (2004). Clinical significance of signet ring cell rectal carcinoma. Int. J. Colorectal. Dis..

[16] Nozoe T., Anai H., Nasu S., Sugimachi K. (2000). Clinicopathological characteristics of mucinous carcinoma of the colon and rectum. J. Surg. Oncol..

[17] Secco G.B., Fardelli R., Campora E., Lapertosa G., Gentile R., Zoli S., Prior C. (1994). Primary mucinous adenocarcinomas and signet-ring cell carcinomas of colon and rectum. Oncology.

[18] Tung S.Y., Wu C.S., Chen P.C. (1996). Primary signet ring cell carcinoma of colorectum: An age-and sex-matched controlled study. Am. J. Gastroenterol..

[19] Li H., Zong Z., Zhou T., Sun L., Wang A., Zhang K., Yi C. (2019). Trends of incidence and survival in patients with gastroenteropancreatic signet ring cell carcinoma: An analysis from the Surveillance, Epidemiology, and End Results program. J. Gastrointest. Oncol..

[20] Nitsche U., Friess H., Agha A., Angele M., Eckel R., Heitland W., Jauch K.W., Krenz D., Nussler N.C., Rau H.G. (2016). Prognosis of mucinous and signet-ring cell colorectal cancer in a population-based cohort. J. Cancer Res. Clin. Oncol..

[21] Yang L.L., Wang M., He P. (2020). Clinicopathological characteristics and survival in colorectal signet ring cell carcinoma: A population-based study. Sci. Rep..

[22] Lin J.Z., Qiu M.Z., Xu R.H., Dobs A.S. (2015). Comparison of survival and clinicopathologic features in colorectal cancer among African American, Caucasian, and Chinese patients treated in the United States: Results from the surveillance epidemiology and end results (SEER) database. Oncotarget.

[23] Shin H.R., Won Y.J., Jung K.W., Kong H.J., Yim S.H., Lee J.K., Noh H.I., Lee J.K., Pisani P., Park J.G. (2005). Nationwide cancer incidence in Korea, 1999~2001; first result using the national cancer incidence database. Cancer Res. Treat..

[24] Benesch M.G., Mathieson A. (2020). Epidemiology of signet ring cell adenocarcinomas. Cancers.

[25] Nam S., Kim D., Jung K., Choi Y.J., Kang J.G. (2020). Analysis of the incidence and clinical features of colorectal nonadenocarcinoma in Korea: A national cancer registry-based study. Ann. Coloproctol..

[26] Hong S., Won Y.J., Park Y.R., Jung K.W., Kong H.J., Lee E.S. (2020). The Community of Population-Based Regional Cancer Registries. Cancer statistics in Korea: Incidence, mortality, survival, and prevalence in 2017. Cancer Res. Treat..

[27] Bray F., Ferlay J., Forman D., Bray F., Brewster D.H., Gombe Mbalawa C., Kohler B., Piñeros M., Steliarova-Foucher E., Swaminathan R. (2014). Age standardization. Cancer Incidence in Five Continents Vol. X, IARC Scientific Publications No. 164.

[28] Ederer F., Heise H. (1959). Instructions to IBM 650 Programmers in Processing Survival Computations.

[29] Arifi S., Elmesbahi O., Amarti Riffi A. (2015). Primary signet ring cell carcinoma of the colon and rectum. Bull. Cancer.

[30] Shi T., Huang M., Han D., Tang X., Chen Y., Li Z., Liu C., Xiang D., Wang T., Chen Y. (2019). Chemotherapy is associated with increased survival from colorectal signet ring cell carcinoma with distant metastasis: A surveillance, epidemiology, and end results database analysis. Cancer Med..

[31] Lee W.-S., Chun H.-K., Lee W.Y., Yun S.H., Cho Y.B., Yun H.-R., Park S.-H., Song S.Y. (2007). Treatment outcomes in patients with signet ring cell carcinoma of the colorectum. Am. J. Surg..

[32] Pande R., Sunga A., LeVea C., Wilding G.E., Bshara W., Reid M., Fakih M.G. (2008). Significance of signet-ring cells in patients with colorectal cancer. Dis. Colon Rectum.

[33] Jayanand S.B., Seshadri R.A., Tapkire R. (2011). Signet ring cell histology and non-circumferential tumors predict pathological complete response following neoadjuvant chemoradiation in rectal cancers. Int. J. Colorectal Dis..

[34] Kim S.-E., Paik H.Y., Yoon H., Lee J.E., Kim N., Sung M.-K. (2015). Sex-and gender-specific disparities in colorectal cancer risk. World J. Gastroenterol. WJG.

[35] Zhao Z., Yan N., Pan S., Wang D.-W., Li Z.-W. (2020). The value of adjuvant chemotherapy in stage II/III colorectal signet ring cell carcinoma. Sci. Rep..

